# Is mesh always necessary in every small umbilical hernia repair? Comparison of standardized primary sutured versus patch repair: retrospective cohort study

**DOI:** 10.1007/s10029-020-02170-1

**Published:** 2020-03-19

**Authors:** K. Mitura, M. Skolimowska-Rzewuska, A. Rzewuska, D. Wyrzykowska

**Affiliations:** 1 Siedlce University of Natural Sciences and Humanities, Faculty of Medical and Health Sciences, Siedlce, Poland; 2 General Surgery Department, Siedlce Hospital, ul. Narutowicza 25, 08-110 Siedlce, Poland

**Keywords:** Umbilical, Hernia, Mesh, Sutured repair, Small, Pain

## Abstract

**Purpose:**

A retrospective analysis was carried out to compare the results of patch repair using ready-made, synthetic mesh (PR) and sutured repair (SR) based on standard protocols. The accumulated recurrence rate was accepted as the primary outcome. Pain at rest and during exercise, cosmetic effect and treatment satisfaction were chosen as the secondary endpoints.

**Methods:**

Adult patients after elective, open surgical repair of a single, primary umbilical hernia < 2 cm in diameter were included. Patients with incarceration or strangulation, after previous umbilical hernia repair or other abdominal surgical interventions were excluded. In the SR group, single-layer sutures were placed using the short-stitch technique. In PR group, a 6.3-mm ready-made Parietene Ventral Patch (Medtronic) was used.

**Results:**

161 patients (104 in PR and 57 in SR groups) were included in the study (22 months follow-up). Nine recurrences were observed [six in PR (5.8%) and three in SR group (5.2%)]. In PR group, three patients (2.9%) reported complaints at rest and none in SR group, while 18 patients (17.3%) in PR group reported pain during exercises and 7 (12.3%) in SR group.

**Conclusion:**

For the smallest umbilical hernias, the use of dense fascia suturing (short-stitch technique) may be an effective alternative to patch repair techniques in patients with no additional risk factors for recurrence. The mesh patch repair method is associated with a significantly higher risk of postsurgical pain. Diastasis recti is a factor favoring umbilical hernia recurrence after both pure tissue repair and patch repair.

## Introduction

The use of mesh in ventral hernia repair is a commonly recognized procedure. An increasing number of studies indicate the need for mesh in the treatment of small umbilical hernias, which has been confirmed by recent guidelines [1]. Studies comparing the sutured repair and the mesh repair of small umbilical hernias indicate a significant reduction in recurrence following the use of synthetic material [2–4]. Nevertheless, surgeons often still opt for sutured repair, particularly in cases of very small hernias [5–7]. Therefore, there is doubt as to whether the uncritical use of mesh in all umbilical hernia cases is justified.

Analysis of the available literature reveals much ambiguity regarding the use of mesh in the repair of small umbilical hernias [8]. The use of 4.2-cm-diameter mesh was reported for the treatment of 2-cm-diameter hernias, which produced only a 1.15-cm margin; this margin is commonly considered to be insufficient for the safe management of the hernial orifice [9]. On the other hand, the use of ready-made patch-type mesh in the treatment of hernias as small as 0.5 cm in diameter has also been reported [10–12]. It is impossible to introduce that type of mesh through such a small orifice, and yet no widening of the orifice was mentioned [13]. At the same time, in sutured repair analyses, no information is usually given regarding the method of suturing, e.g., continuous, interrupted, density, margin, absorption, direction, etc. [14–16]. There is often no information regarding the accompanying diastasis recti [17]. All such methodological doubts make it difficult to assess how a hernia has been repaired and whether all methods of sutured repair offer similar results.

For this reason, this retrospective analysis was carried out to compare the results of patch repair using ready-made, synthetic mesh and sutured repair based on standard protocols. The cumulative recurrence rate was accepted as the primary outcome. Pain at rest and during exercise, cosmetic effect and treatment satisfaction were chosen as the secondary endpoints.

## Materials and methods

### Patients

Adult patients who underwent the elective, open surgical repair of a single, only primary, symptomatic umbilical hernia were included in the retrospective cohort study. Patients with incarceration or strangulation who underwent urgent surgical repair were excluded. Patients with incisional hernia were excluded. Patients with a history of previous umbilical hernia repair were excluded. Patients who underwent laparoscopic repair were excluded. Patients who underwent other abdominal surgical interventions after the umbilical hernia repair were excluded. Patients lost to follow-up (no contact, change of address, death) and patients not giving consent for participation in the study were excluded. Only patients with a hernial orifice less than 2 cm in diameter were analyzed.

All patients who underwent surgery between December 1st, 2015, and November 30th, 2019, were considered. The follow-up period was defined as the time from the surgical repair to recurrence or to the end of study date on December 31st, 2019.

### Qualifications in the study

The final decision on the choice of surgical method was made by the operating surgeon. Prior to surgery the patient signed an informed consent allowing the operating surgeon to decide on the type of repair performed depending on the intraoperative circumstances. The form has been prepared according to the recommendations of a national surgical society. In the absence of combination of additional risk factors, including obesity or overweight, diastasis recti, the presence of other hernias, diabetes, smoking, and a history of surgical interventions in the vicinity of the umbilicus, a subjective decision for sutured repair was usually made [18]. Sutured repair was also performed in the case of a very narrow hernial orifice (< 1 cm), associated with the need to make the orifice wider to accommodate the mesh.

### Procedure

All surgical procedures were performed under general anesthesia. Before the procedure, patients were given antibiotic prophylaxis, and the final choice of the surgical method was made by the surgeon during the course of the procedure. Drains were not used. No surgery involved widening of the hernial orifice.

An arched skin incision approximately 3–4 cm in length was made along the lower edge of the umbilicus. Then, the hernial sac was isolated and separated from the skin. The anterior lamella of the fascia was isolated and separated from subcutaneous adipose tissue over the radius of approximately 2 cm. In every case, the hernial sac was opened and its content was controlled. After the fascia was repaired, the subcutaneous tissue was sutured with transverse sutures cranially and caudally to the umbilicus, and the skin at the bottom of the umbilicus was anchored with a single suture to the fascia, thus recreating the bowl shape of the umbilicus.

### Sutured repair

After dissecting the edges of the fascia, continuous sutures with prolonged absorption characteristics (MonoMax 0 or MonoPlus 0; *B. Braun*) were used. The fascia was sutured longitudinally, starting from the suture line 10 mm above the hernial orifice and ending it at the same distance below the orifice. Single-layer sutures were placed using the short-stitch technique, with a lateral margin of 5–7 mm and a distance of 4–6 mm between subsequent needle passages.

### Patch repair

Mesh repair involved the use of the 6.3-cm-diameter Parietene Ventral Patch (*Medtronic*). As this polyester mesh is coated with collagen as the anti-adhesive layer, it may be placed intraperitoneally. Intraperitoneal mesh placement was used for all patients enrolled in the study who underwent mesh repair. Before mesh placement, the possible presence of intraperitoneal adhesions around the hernial orifice was controlled, and any adhesions present were excised. In patients with a thickened and rigid umbilical or sickle fold, the fold was separated from the linea alba to obtain a flat surface for mesh adherence. The mesh was equipped with two semicircular, rigid rings to facilitate its positioning. Four poles of the mesh were fixed to the fascia with single nonabsorbable sutures (Optilene 0; *B. Braun*). Then, the fascia was sutured using the same technique as that described for the sutured repair technique.

### Outcomes

All operated patients provided information on their treatment through a phone questionnaire. Recurrence was the primary outcome. Patients reporting existing or suspected recurrence were additionally examined by an experienced surgeon and underwent a supplementary ultrasound examination. The presence of pain, determined according to the verbal rating scale (VRS; none 1; mild 2; moderate 3; severe 4), the cosmetic effect, and satisfaction with the received treatment were the secondary outcomes. Additionally, sex, BMI, the duration of the surgical procedure and the presence of postsurgical complications were determined based on medical files.

### Statistical methods

Data are expressed as the mean ± standard deviation (SD) and range. *P* values < 0.05 were assumed statistically significant. Data were analyzed using Statistica 11.0 software (*StatSoft*). Continuous values were analyzed by *t* test, and Pearson’s Chi squared test was used for categorized values.

## Results

**Fig. 1:**
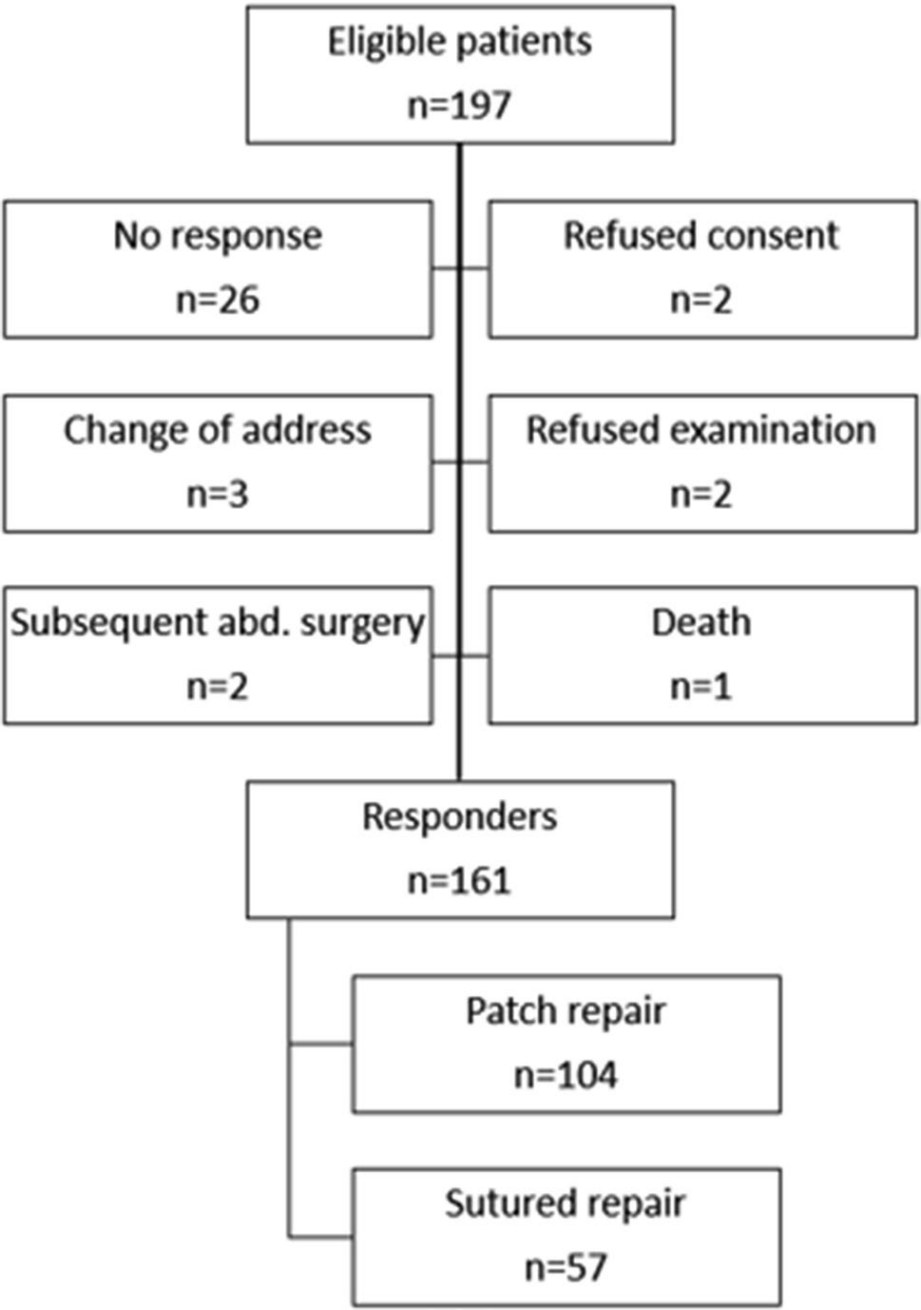
Study flow chart

The characteristics of the patients are given in Table 1. In all, 197 patients were operated on during the analyzed 4-year period, and 161 patients (57 underwent sutured repair and 104 underwent patch repair) who responded to the questionnaire (response ratio: 86%) were included in the study. A total of 26 patients were excluded as they were lost to follow-up (Fig. 1). The mean postsurgical follow-up period was 22 months (range 1–49 months).

**Table 1:** Patient characteristics

	Sutured repair	Mesh repair	All	*p*
Number of patients (%)	57 (35.4)	104 (64.6)	161 (100)	
Age (years)	46.9 (21–85)	49.2 (25–77)	48.4 (21–85)	0.053
Females/males (%)	21/36 (36.8/63.2)	39/65 (37.5/62.5)	60/101 (37.3/62.7)	0.174
Hernia size (cm)	1.3 (0.5–2.0)	1.6 (1.0–2.0)	1.5 (0.5–2.0)	0.302
BMI (kg/m^2^)	28.1 (17.7–38.6)	30.2 (20.8–41.1)	29.5 (17.7–41.1)	0.061
Smokers (%)	7 (12.3)	31 (29.8)	38 (23.6)	**< 0.001**
Diabetes (%)	2 (3.2)	9 (8.7)	11 (6.8)	**0.038**
Clinical signs of diastasis recti (%)	7 (12.3)	40 (38.4)	47 (29.2)	**< 0.001**
Presence of other hernias (%)	2 (3.2)	9 (8.7)	11 (6.8)	**0.019**

Statistically significant difference with the *p* value of < 0.05 are marked in bold

### Primary outcome

In total, nine cases of recurrence were observed (5.6%); there were three cases of recurrence in the sutured repair group (5.2%) and six cases of recurrence in the patch repair group (5.8%) (Table 2). In both groups, recurrence occurred in overweight and obese patients (mean BMI 30.8, range 26.2–41.1). In the sutured repair group, recurrence occurred only in the group of patients with coexistent diastasis recti. The mean period between the surgical procedure and recurrence was 16 months (9–39).

**Table 2:** Characteristics of recurrence

	Sutured repair *n* = 62	Mesh repair *n* = 119	All *n* = 181	*p*
Cases of recurrence (%)	3 (4.8)	6 (5.0)	9 (5.0)	0.739
Age (years)	49.7 (36–64)	45.8 (35–70)	47.1 (35–70)	0.064
Females/males (%)	0/3 (0/100)	2/4 (33.3/66.7)	2/7 (22.2/77.8)	0.159
BMI (kg/m^2^)	27.3 (26.2–37.4)	31.8 (20.8–41.1)	31.2 (26.2–41.1)	**0.028**
Clinical signs of diastasis recti (%)	3 (100)	4 (66.7)	53 (77.8)	**0.039**
Time to recurrence (months)	14 (9–26)	17 (10–39)	16 (9–39)	0.370

Statistically significant difference with the *p* value of < 0.05 are marked in bold

### Secondary outcomes

In the patch repair group, three patients (2.9%) reported complaints at rest, and 18 patients (17.3%) reported pain during intensified physical exercise. In the sutured repair group, no patient reported pain at rest, but 7 (12.3%) reported discomfort associated with movement (pulling or prickling) (Table 3). Only four patients (3.8%) in the patch repair group and two (3.5%) in the sutured repair group were dissatisfied with the aesthetic outcome of the surgical procedure (*p* = 0.412). Nevertheless, the vast majority of patients ranked their satisfaction with the treatment as very high or high (94.2% in the patch repair group and 93.0% in the sutured repair group; *p* = 0.606). None of the patients reported dissatisfaction with the received treatment.

**Table 3:** Characteristics of complaints and patient satisfaction

	Sutured repair *n* = 57	Mesh repair *n* = 104	All *n* = 161	*p*
Pain at rest (none/mild/moderate/severe)	57/0/0/0	101/0/1/2	158/0/1/2	**0.015**
Pain during physical activity (none/mild/moderate/severe)	50/5/2/0	86/8/5/5	136/13/7/5	0.086
Assessment of cosmetic effect (very good/good/moderate/ none)	34/19/2/2	50/46/4/4	84/65/6/6	0.412
Satisfaction with the treatment (very high/high/moderate/none)	30/23/4/0	59/39/6/0	59/39/6/0	0.606

Statistically significant difference with the *p* value of < 0.05 are marked in bold

## Discussion

Treatment failure in cases of even small umbilical hernias may lead to recurrence. The anterior abdominal wall becomes deformed, the hernial sac increases, and repeated repair surgery is necessary, which is more difficult, more complicated and carries a higher risk of another recurrence. For this reason, neglecting the problem of small hernias may cause dramatic consequences for the patient and lead to the necessity of complex treatment in the future [2].

As such, among other reasons, in the last decade, the view that synthetic mesh needs to be used even in the repair of very small hernias has been promoted to avoid troublesome recurrence. However, despite recent guidelines, there is still no consensus among surgeons as to whether very small umbilical hernias, e.g., those less than 1 cm in diameter, must be reinforced with mesh. This dilemma stems mainly from the technical aspects of such repair. The placement of mesh several centimeters in diameter in the retromuscular, pre- or intraperitoneal space is difficult through such a narrow orifice, and particularly in obese patients, there is often doubt regarding whether flat mesh has been placed correctly. Many reports mentioning the sublay method do not provide information regarding orifice widening in cases of very small hernias [11–13, 15]. Therefore, we are faced with another question: what was the actual size of the hernia—the primary size or that following widening? In particular, shape memory mesh requires a larger orifice through which it is introduced [20]. An attempt to place coated mesh through a narrow orifice may lead to damage to the nonadhesive layer, the inability to fit the mesh rings through the tight hole, and the lack of complete control over the margins and alignment of the mesh, which can promote adhesions and bending of the mesh in half, similar to a ‘clamshell’ or ‘potato chip’ mechanism, and thus favoring recurrence [21].

On the other hand, the introduction of flat mesh into the retromuscular space is associated with the need to cut the medial edges of the posterior lamellae of the rectus muscle sheaths—a procedure that is also technically challenging through a narrow orifice [22]. All these aspects cause some surgeons to believe that to place the mesh, it is necessary to dissect an appropriate space, damaging natural structures (e.g., the linea alba, lamellae of rectus muscle sheaths). This is one of the reasons why they decide to perform the least invasive hernia treatment method, i.e., simple sutures.

Unfortunately, an optimal technique for hernial orifice closure has not been defined. Numerous methods are in use, including single sutures, figure-eight sutures, letter-Z sutures, and continuous sutures, with single or double suture layers, allowing longitudinal and transverse fascia edge approximation with single or double tissue layers. The Mayo method (‘vest over pants’) has been used as a standard technique for many years, involving transverse double-layer suturing of the fascial edges [19]. Various suture materials have also been used, including short-, medium-, and long-term absorbable sutures, as well as nonabsorbable sutures, with a range of thicknesses [14–16]. Additionally, the number of sutures and the distance between needle passages depends on the preference of the operating surgeon. This great diversity of the methods used means that the results of treatment may differ significantly; thus, there is significant variation in the rate of recurrence after sutured repair was reported in the literature (3–21%) [1, 2, 4–8, 11, 13–15]. The suturing standard based on the short-stitch technique was used in the presented paper, meaning that dense passages of long-term absorbable continuous sutures were used [23]. We believe that the dense suture weave results in an even tension distribution along the seam line, reduces ischemia associated with tissue compression by sutures, and allows the creation of solid fusion.

It is worth noting that in cases of inguinal hernias, also after a period of infatuation with tension-free techniques, an optimal group of patients is currently being sought for whom similar treatment effects can be achieved with pure tissue repair techniques [24]. This is important because there is a growing group of patients who refuse to undergo mesh repair. In cases of inguinal hernias, Köckerling et al. noted that the Shouldice method offers similar treatment effects as the Lichtenstein method in cases of small oblique hernias in young and slim patients [24]. In our study material, sutured repair was also used in patients with a lower BMI, without associated burdens, and the surgery was performed in cases of small hernias. The rate of recurrence was similar in patients with and without mesh and was not significantly different. At the same time, in the sutured repair group, less pain was observed both at rest and during exercise, although only in the latter case a statistically significant difference was noted.

Complete disqualification of the sutured repair method in cases of small hernias is inconsistent with the procedure applied in closing wounds after laparoscopic procedures in the umbilical region. These cases also involve fascia incisions of 10–20 mm in diameter, which are initially closed with sutures. No indications for the preventive use of synthetic mesh in such cases have been established so far, as it is in specific laparotomy procedures for abdominal aortic aneurysm or ostomy creation [25, 26]. It may be indirectly concluded that primary repair works in most patients after all.

Undoubtedly, aside from obesity and overweight, the coexistence of diastasis recti is a risk factor for recurrence, especially in men over 40 years of age [1, 27]. The use of mesh repair instead of sutured repair seems reasonable. In the presented study material, all cases of recurrence observed after sutured repair occurred in this particular group of patients.

Another factor affecting the onset of recurrence is the development of postoperative complications. Both seromas and hematomas may increase the risk of surgical site infection [3, 5]. For this reason, the same scheme of applying triclosan-coated sutures was used each time [23]. After the repair, the subcutaneous tissue was transversely approximated in the cranial direction from the umbilicus. Then, the bottom of the umbilicus was fixed with an absorbable suture transverse to the fascia, and the subcutaneous tissue was once again approximated transversely in the caudal direction from the umbilicus. This arrangement of sutures resulted in complete closure of the subcutaneous tissue and elimination of the empty space, thus reducing fluid collection.

The main weakness of this retrospective analysis is the selection bias. However, the selected two groups of patients were comparable in terms of their age, sex, hernia size and BMI (no significant differences). Nevertheless, those groups still differ significantly in terms of smoking incidence, diabetes, concomitant hernias and diastasis recti. But, as it has been stated above, these additional risk factors commonly obliged the surgeon to introduce the patch repair rather than suture repair. We believe that future prospective study with randomized group allocation may provide definite answers. However, our results imply that this issue ought to be thoroughly studied in the following analyses to verify the predisposition we have noticed in our study.

The discussion about the possibility of using sutured repair in the treatment of small umbilical hernias is important because we are currently witnessing a bloom of minimally invasive abdominal hernia repair techniques, including TARUP, eTEP, TAPP, and eRS [28, 29]. It seems that before the popularization of these attractive techniques, it is important to set indications and boundaries for the use, location and size of synthetic materials. The purpose of this should be to prevent the excessive use of some irreversible techniques in cases of very small hernias without additional risk factors and to avoid overuse of these methods.

## Conclusions

For the smallest umbilical hernias, the use of dense fascia suturing based on the assumptions of the short-stitch technique may be an effective alternative to patch repair techniques in patients with no additional risk factors for recurrence. The mesh patch repair method is associated with a significantly higher risk of postsurgical pain. Diastasis recti is a factor favoring umbilical hernia recurrence after both pure tissue repair and patch repair.
